# Application of volume displacement technique in the operation of breast dermatofibrosarcoma protuberans: A case report

**DOI:** 10.1097/MD.0000000000045188

**Published:** 2025-10-17

**Authors:** Sen Li, Yingfang Shi, Zhichun Wang

**Affiliations:** a Breast Surgery, Jiujiang City Key Laboratory of Cell Therapy, JiuJiang NO.1 People’s Hospital, Jiujiang, Jiangxi, PR China.

**Keywords:** breast tumor, dermatofibrosarcoma protuberans, immunohistochemistry, oncoplastic surgery, rare tumor, reconstruction, volume displacement technique, wide local excision

## Abstract

**Rationale:**

Dermatofibrosarcoma protuberans (DFSP) of the breast is exceedingly rare. Surgical management requires achieving negative margins while preserving breast aesthetics, which presents a significant challenge. This case demonstrates the use of the volume displacement technique as a reconstructive approach to balance oncologic safety with cosmetic outcomes.

**Patient concerns:**

A 41-year-old woman was referred for evaluation of a palpable mass in the left breast, identified during routine physical examination. She reported no pain or systemic symptoms.

**Diagnoses:**

Imaging revealed a subcutaneous mass with mixed echogenicity and abundant vascularity. Core biopsy suggested a spindle cell tumor. Histopathology and immunohistochemistry confirmed the diagnosis of DFSP (CD34 positive, Vimentin positive, negative for other markers).

**Interventions:**

The patient underwent wide local excision with negative margins under general anesthesia, followed by immediate reconstruction using the volume displacement technique to preserve breast contour.

**Outcomes:**

Postoperative pathology confirmed complete tumor removal with negative margins. The patient recovered without complications, and no recurrence was observed during follow-up. Cosmetic results were satisfactory.

**Lessons:**

DFSP of the breast is rare, and achieving oncologic safety without compromising aesthetics is challenging. The volume displacement technique offers a valuable reconstructive option, allowing wide excision while maintaining breast contour. This approach may serve as a useful strategy in similar cases, though further studies are needed to confirm its broader applicability and long-term outcomes.

## 1. Introduction

Dermatofibrosarcoma protuberans (DFSP) is a rare soft tissue tumor that can occur in various locations, including the breast. Surgical resection with clear margins is the primary treatment modality for DFSP.^[1]^ However, achieving adequate oncological margins while preserving cosmesis in breast surgery can be challenging.^[2]^ Here, we present a case report of a patient with DFSP of the breast successfully managed using the volume displacement technique. This technique allows for tumor resection with immediate breast reconstruction, minimizing deformity and preserving cosmesis.^[3]^ The patient underwent wide local excision followed by immediate reconstruction using a volume displacement technique with favorable oncological and cosmetic outcomes. Our case highlights the efficacy of the volume displacement technique in achieving both oncological clearance and aesthetic results in breast surgery for DFSP. According to our literature review, we did not find any relevant reports before us describing the use of volume displacement techniques specifically for DFSP of the breast.

## 2. Case presentation

The patient, a 41-year-old female, was admitted to the hospital after detecting a left breast mass during a routine physical examination that had been present for over 4 months. Her past medical history was unremarkable, with no history of breast disease, prior surgery, or systemic illness. Family history revealed no known malignancies, and she reported no relevant psychosocial stressors.

On physical examination, no significant enlargement of superficial lymph nodes was noted. The breasts were symmetrical, with no peau d’orange appearance. The nipples and areolas were unremarkable, showing no scaling or discharge. A palpable, well-defined, firm, and mobile mass measuring ~2.5 cm × 1.0 cm was identified 3 cm from the nipple at the 10 o’clock position of the left breast. No abnormalities were detected in the right breast.

**Figure 1.**
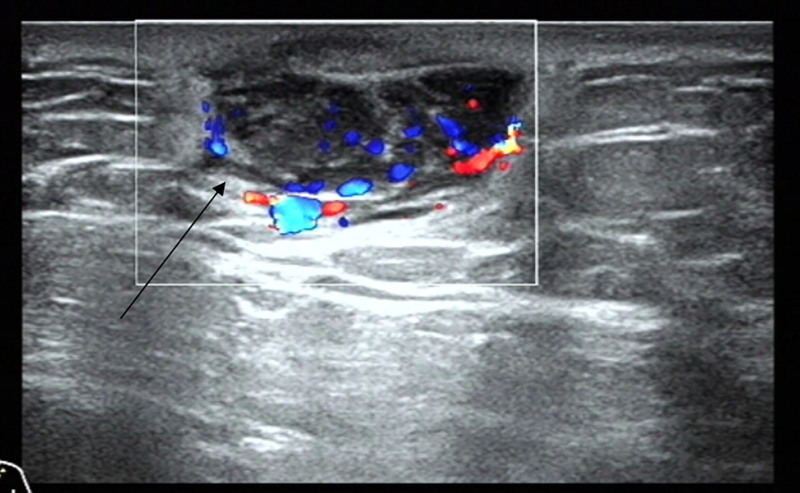
Ultrasound of the left breast showed a mixed echogenic mass in the subcutaneous fat layer of the left breast with rich blood supply.

On January 3, 2024, bilateral breast and lymph node color Doppler ultrasound revealed a mixed echoic mass located in the subcutaneous fat layer of the left breast, demonstrating abundant vascularity and an increase in size compared with the previous ultrasound performed on July 4, 2023. The lesion was classified as breast imaging-reporting and data system category 3 (Fig. 1).

**Figure 2.**
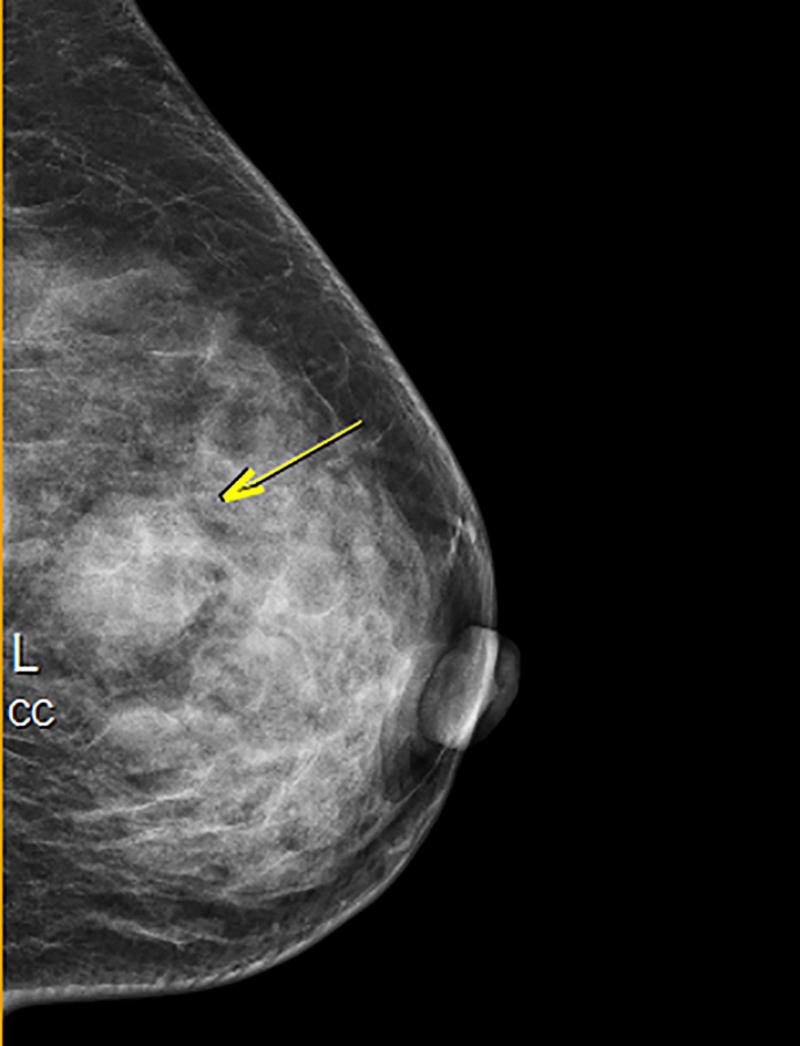
Mammography combined with physical examination showed a subcutaneous nodule in the middle and upper part of the left breast, which was considered to be sebaceous adenoma. BI-RADS class 4A. BI-RADS = breast imaging-reporting and data system.

On January 4, 2024, bilateral breast mammography showed a subcutaneous nodule in the upper central region of the left breast, suggestive of a possible sebaceous adenoma. The lesion was categorized as breast imaging-reporting and data system 4A (Fig. 2).

Following preoperative evaluation, no contraindications to surgery were identified. On January 5, 2024, the patient underwent excisional biopsy of the left breast lesion under local anesthesia. Intraoperative frozen section analysis indicated a spindle cell tumor, pending further histopathological and immunohistochemical evaluation.

**Figure 3.**
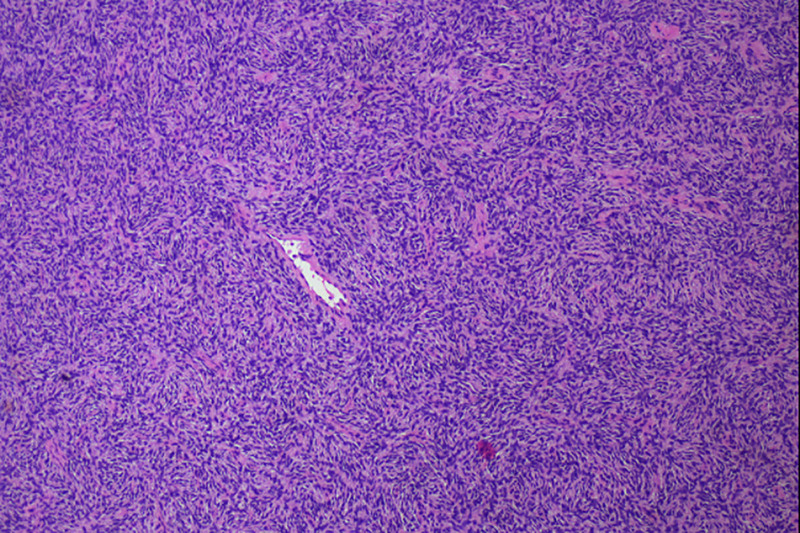
Pathology results-(hematoxylin and eosin [H&E] ×40) shows a uniform distribution of spindle-forming fibroblasts arranged in a typical storiform pattern with a fibrous matrix background.

On January 12, 2024, the final pathology report confirmed the diagnosis of DFSP of the left breast. Immunohistochemical staining revealed the following results: SRY-box transcription factor 10 (−), β-catenin (−), Ki-67 antigen (~10% positive), P53 (complete loss), CD117 (−), smooth muscle actin (−), Desmin (−), S-100 protein (−), cluster of differentiation 34 (+), and Vimentin (type III intermediate filament protein) (+) (Fig. 3). These findings were consistent with DFSP.^[4,5]^

**Figure 4.**
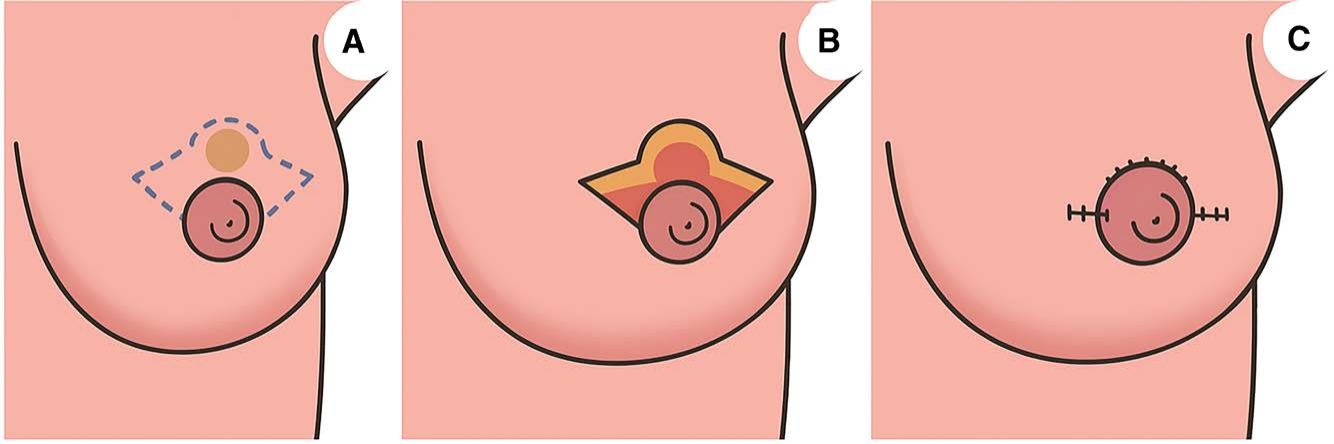
Schematic diagram of the surgical method: the dashed line in the figure represents the surgical incision, and the wavy line represents the surgical incision after suture.

Subsequently, on January 16, 2024, wide local excision of the left breast skin tumor was performed under general anesthesia, combined with fascial tissue flap reconstruction using a volume displacement technique (Fig. 4). The goal was to achieve oncological safety while maintaining breast contour and cosmesis.^[6,7]^ The operation was carried out after the approval of the Ethics Committee and the Medical Department of the First People’s Hospital of Jiujiang. The patients signed the informed consent after being fully informed.

On January 19, 2024, final pathological examination demonstrated: (1) DFSP of the left breast accompanied by granulation tissue proliferation and multinucleated giant cell reaction and (2) clear surgical margins in all directions (upper inner, lower inner, upper outer, and lower outer quadrants), with no residual tumor detected.

**Figure 5.**
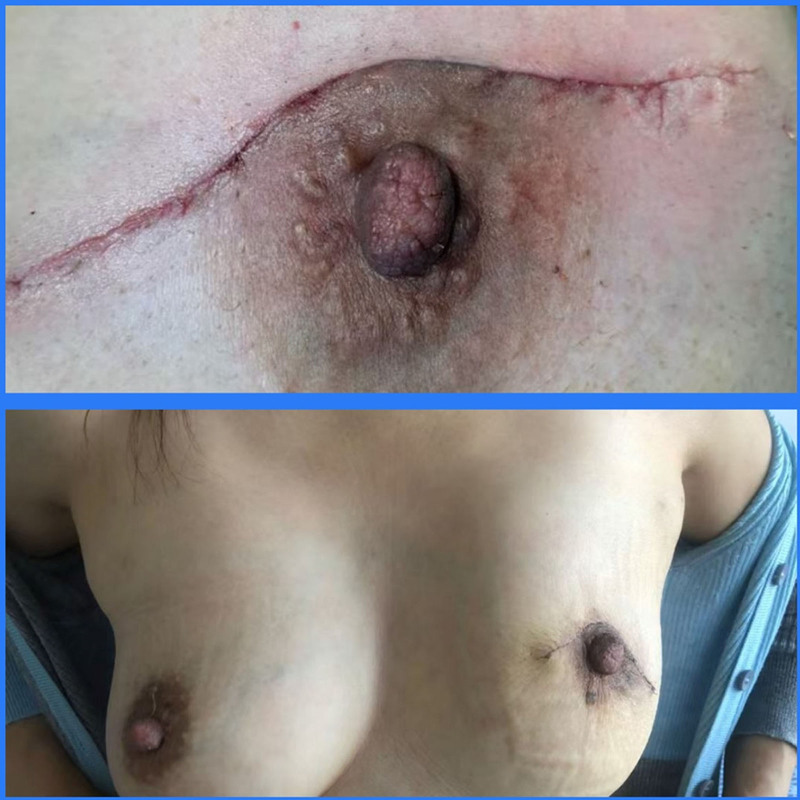
Photographs of patients 1 and 2 weeks after surgery. Postoperative outcomes following volume displacement reconstruction of the left breast. The image demonstrates good breast symmetry, preservation of contour, and minimal scarring. No deformity, skin retraction, or nipple-areolar complex displacement are evident, highlighting the cosmetic advantage of this technique.

The patient recovered well after surgery. At the 1- and 2-week follow-up, the surgical site demonstrated satisfactory wound healing without infection, hematoma, or seroma. The breast contour remained symmetrical with the contralateral side, and only a minimal linear scar was visible at the resection site. Functional outcomes were preserved, and the patient reported high satisfaction with both oncological safety and cosmetic appearance (Fig. 5).

She has been followed up for 12 months with no evidence of recurrence or complications, as assessed by physical examination and ultrasound.

## 3. Discussion

DFSP is a low-grade malignant tumor arising from the dermis and subcutaneous tissue. It accounts for ~1.8% to 6.0% of all soft tissue sarcomas and is characterized by slow growth, aggressive local invasion, a high postoperative recurrence rate, and low potential for distant metastasis.^[1,2]^ Clinically, DFSP often presents as a firm, protuberant, skin-colored, or violaceous nodule, sometimes accompanied by ulceration or hyperpigmentation of the surrounding skin.^[3]^ The tumor typically affects young to middle-aged adults between 20 and 50 years of age, with a slight male predominance.^[4]^ The most common anatomical sites include the trunk, proximal extremities, head, and neck, while involvement of the breast is exceedingly rare.^[5,6]^ Recent reports also emphasize that breast DFSP may present with subtle features mimicking benign lesions, making accurate preoperative diagnosis even more challenging.^[7]^

At initial presentation, DFSP may be misdiagnosed as a benign lesion and inadequately excised without sufficient margins, leading to recurrence rates ranging from 24% to 60%.^[5]^ Its infiltrative growth pattern and absence of a true capsule contribute to these high recurrence rates if not managed appropriately.^[8]^

Surgical resection remains the primary treatment modality for DFSP. Wide local excision and Mohs micrographic surgery are commonly used. In breast surgery, however, surgeons must balance oncological safety with cosmetic preservation.^[9]^ Achieving negative margins while preserving breast contour and function presents a particular challenge in breast DFSP.

In general, surgical guidelines for DFSP recommend excision with margins extending 3 to 5 cm beyond the tumor borders, with resection extending 1 to 2 cm deeper than the normal subcutaneous tissue to achieve adequate clearance.^[10]^ Axillary lymph node dissection is typically unnecessary due to the tumor’s low propensity for nodal metastasis.^[11]^ Postoperative radiotherapy may serve as an adjuvant treatment option to reduce recurrence, especially when negative margins are difficult to obtain.^[12]^ In unresectable, advanced, or metastatic cases, targeted therapy with imatinib mesylate, a tyrosine kinase inhibitor, has demonstrated efficacy through inhibition of the PDGFB fusion gene pathway commonly involved in DFSP pathogenesis.^[13,14]^

In our case, given the considerable skin excision required for oncological clearance, the volume displacement technique was utilized to achieve both adequate tumor resection and immediate reconstruction. One of the primary advantages of this technique is that it allows for immediate breast reshaping without the need for staged reconstructive procedures, thereby reducing the psychological burden associated with delayed cosmetic correction and minimizing operative morbidity.^[15]^ Moreover, the technique preserves breast sensation and function by minimizing disruption to the surrounding tissues, contributing to improved quality of life and patient satisfaction.^[16]^ This advantage has been highlighted in recent reconstructive series, where volume displacement approaches were associated with higher patient satisfaction and fewer complications compared with more invasive techniques.^[17]^

Compared with alternative strategies such as volume replacement techniques, staged reconstruction, or even mastectomy with delayed reconstruction, the volume displacement method is less invasive, avoids the need for foreign implants, and better maintains breast symmetry in selected patients. However, it may be limited in cases of large tumor-to-breast ratio, where cosmetic outcomes may be suboptimal.

From an oncological perspective, achieving clear surgical margins is paramount to minimizing local recurrence in DFSP. The volume displacement technique enables wide local excision while maintaining adequate margins, as demonstrated in our case. Close multidisciplinary collaboration between oncologic and reconstructive surgeons is essential to optimize both oncologic safety and cosmetic outcomes.^[18]^

Nonetheless, certain limitations exist for the volume displacement technique, including the potential for incomplete resection or suboptimal aesthetic outcomes in select cases. Careful patient selection, consideration of tumor characteristics, and surgeon expertise remain critical factors influencing the success of this approach.^[19]^

Importantly, this case also suggests that the volume displacement technique may serve as a reference for future surgical practice in rare breast sarcomas. Larger case series and multi-institutional studies have been called for to validate the oncological safety and long-term cosmetic outcomes of such approaches.^[17]^

In conclusion, the volume displacement technique offers a valuable surgical option for managing DFSP of the breast, balancing oncologic clearance with preservation of cosmesis. Recent studies on surgical oncology have also highlighted the importance of prognostic indices, such as the Prognostic Nutritional Index, in predicting postoperative complications across different malignancies.^[20]^ Incorporating such assessments may further refine patient selection and perioperative management in DFSP cases requiring complex reconstruction. This report is limited by its single-case nature, which restricts generalizability. Moreover, longer follow-up is necessary to confirm the durability of both oncological and cosmetic outcomes. Larger multicenter series are required to validate these finding. Further studies are warranted to evaluate long-term outcomes and to compare the efficacy of various surgical techniques for this rare but challenging condition.

## 4. Conclusion

DFSP of the breast is exceedingly rare. The volume displacement technique offers a safe and effective surgical option for achieving both oncological clearance and aesthetic preservation. Our case demonstrates favorable oncological and cosmetic results with 12 months of recurrence-free survival. Further studies with larger cohorts are needed to establish long-term oncologic and cosmetic outcomes.

## Author contributions

**Conceptualization:** Sen Li.

**Data curation:** Sen Li.

**Formal analysis:** Sen Li.

**Funding acquisition:** Yingfang Shi.

**Investigation:** Yingfang Shi.

**Methodology:** Yingfang Shi.

**Supervision:** Zhichun Wang.

**Visualization:** Zhichun Wang.

**Writing – original draft:** Sen Li.

**Writing – review & editing:** Zhichun Wang.
